# Assessing Potential Valve-Preserving Effects of SGLT2 Inhibitors in Degenerative Aortic Stenosis: A Propensity-Matched Study

**DOI:** 10.3390/jcm15020714

**Published:** 2026-01-15

**Authors:** Olivier Morel, Michael Guglieri, Antonin Trimaille, Benjamin Marchandot, Arnaud Bisson, Amandine Granier, Valérie Schini-Kerth, Anne Bernard, Laurent Fauchier

**Affiliations:** 1 Department of Cardiovascular Medicine, Nouvel Hôpital Civil, Strasbourg University Hospital, 67098 Strasbourg, France; antonin.trimaille@gmail.com (A.T.); amandine.granier@chru-strasbourg.fr (A.G.); 2 UR 3074 Translational Cardiovascular Medicine, CRBS, University of Strasbourg, 67000 Strasbourg, France; 3 GERCA Groupe pour l’Enseignement, la Prévention et la Recherche Cardiologique en Alsace, 67200 Strasbourg, France; 4 Hanoï Medical University, Hanoi 100000, Vietnam; 5 Service de Cardiologie, Centre Hospitalier Universitaire Trousseau et Faculté de Médecine, Université François Rabelais, 37000 Tours, France; m.guglieri@chu-tours.fr (M.G.); laurent.fauchier@univ-tours.fr (L.F.); 6 Inserm U1327 ISCHEMIA “Membrane Signalling and Inflammation in Reperfusion Injuries”, Université de Tours, 37000 Tours, France

**Keywords:** aortic valve, TAVR, SAVR, TAVI, degenerative aortic stenosis, gliflozins, empaglifozin, dapaglifozin

## Abstract

**Highlights:**

**What is the clinical question being addressed?**

- Among patients with aortic stenosis, is SGLT2 inhibitor therapy associated with differences in key clinical outcomes, including all-cause mortality and progression to aortic valve replacement?

**What is the main finding?**

- In patients with aortic stenosis, SGLT2 inhibitor therapy was associated with lower risks of mortality and aortic valve replacement (SAVR or TAVR). These observations raise the possibility of a favorable effect on the clinical trajectory of aortic stenosis; however, the findings should be interpreted as associative given the absence of systematic longitudinal assessment of valvular disease progression.

**Abstract:**

**Background:** Sodium–glucose cotransporter 2 inhibitors (SGLT2 inhibitors), initially developed for glycemic control in type 2 diabetes, have demonstrated robust cardiovascular and renal benefits. Emerging evidence suggests that these agents may also affect valvular pathobiology, particularly in degenerative aortic stenosis (AS), through anti-inflammatory and antifibrotic mechanisms. **Objectives:** This study evaluated whether SGLT2 inhibitor use is associated with improved clinical outcomes in degenerative AS, including all-cause mortality and the need for SAVR or TAVR, recognizing that these endpoints represent surrogate rather than direct measures of valve hemodynamic progression. **Methods:** A retrospective cohort analysis was conducted using TriNetX, a federated electronic medical record-based research network. Diagnoses are captured using ICD-9/ICD-10-CM codes and medications using ATC codes. Adults with non-rheumatic AS were stratified by SGLT2 inhibitors use. Propensity score matching (1:1) was performed to balance baseline characteristics between treated and untreated groups (n = 10,912 per group). Primary outcomes included all-cause mortality, TAVR, and SAVR during follow-up. Echocardiographic parameters (AVA, Vmax, mean gradient) were not systematically available. **Results:** After adjustment for comorbidities, SGLT2 inhibitor use was independently associated with lower all-cause mortality (6.15% vs. 9.34% HR 0.595; 95% CI 0.552–0.641; *p* < 0.001), TAVR (2.81% vs. 2.89% HR 0.835; 95% CI 0.746–0.934; *p* = 0.002), SAVR (1.28% vs. 1.90% HR 0.514; 95% CI 0.442–0.599; *p* < 0.001), cardiac arrest (0.82% vs. 1.21% HR 0.71; 95% CI 0.582–0.867; *p* < 0.001), and end-stage kidney disease (0.40% vs. 1.0% HR 0.292; 95% CI 0.222–0.384; *p* < 0.001). Although these associations may suggest slower disease progression, interpretation is limited by the lack of systematic echocardiographic follow-up. **Conclusions:** In addition to their established benefits in heart failure and renal protection, SGLT2 inhibitors may have valve-preserving effects in degenerative AS. Because true hemodynamic progression could not be evaluated, these results should be viewed as associations with surrogate clinical endpoints. Prospective studies with standardized imaging are required to determine whether SGLT2 inhibition can directly alter the course of this currently untreatable disease

## 1. Introduction

Sodium–glucose cotransporter 2 inhibitors (SGLT2 inhibitors), initially developed for the treatment of type 2 diabetes mellitus, have shown significant cardiovascular benefits, particularly among patients with heart failure [1]. Beyond their glucose-lowering effects, SGLT2 inhibitors exert a range of pleiotropic actions that may also be relevant to valvular heart disease [2]. Although the expression of SGLT2 in vascular tissues was until recently uncertain, emerging cardiovascular research has clearly demonstrated that subclinical inflammation acts as a key driver of SGLT2 overexpression through activation of the AT1R/NADPH oxidase pathway within human cardiovascular tissues. Mechanistic studies suggest that the cardioprotective effects of SGLT2 inhibition are mediated through improvements in endothelial function, suppression of proinflammatory signaling, and inhibition of profibrotic and procalcific mediators [3,4]. These mechanisms may help slow the progression of degenerative calcific aortic stenosis (AS), a condition for which no effective medical therapy currently exists [5]. Histopathological analyses have identified SGLT2 expression within human calcified aortic valves, further supporting a potential pathophysiological link [4]. Moreover, SGLT2 expression is upregulated in the myocardium, particularly in patients with low-flow, low-gradient (LF/LG) AS, where it correlates with oxidative stress, myocardial fibrosis, and inflammation [6]. In our recent investigations of aortic stenosis, we demonstrated that plasma from patients with severe AS exhibits a distinct proinflammatory phenotype characterized by elevated cytokine levels and increased factor Xa (FXa) activity [7]. This plasma environment markedly enhances oxidative stress in valvular endothelial cells (VECs), leading to endothelial dysfunction via SGLT2 overexpression and promoting a prothrombotic, proinflammatory, and proadhesive phenotype—an effect significantly attenuated by empagliflozin, a selective SGLT2 inhibitor [7]. Collectively, these findings support the concept that SGLT2 inhibitors may exert both cardioprotective and valvuloprotective effects, providing a promising therapeutic avenue in diseases previously considered refractory to medical intervention.

In this context, we aimed to evaluate the associations between SGLT2 inhibitor use and clinical outcomes—including all-cause mortality, aortic valve replacement (both surgical [SAVR] and transcatheter [TAVR]), and cardiovascular events—in a large real-world cohort of patients with aortic stenosis. Because imaging-based measures of AS severity were not systematically available, the analysis focuses on clinical endpoints that may reflect differences in disease trajectory but cannot directly quantify hemodynamic progression.

## 2. Materials and Methods

All statistical analyses were performed on the TriNetX platform. TriNetX is a global federated health research network that provides access to electronic medical records (EMRs) from participating 147 health care organizations in 18 countries, covering approximately 150 million individuals. Inclusion criteria consisted of non-rheumatic aortic valve stenosis (ICD-10-CM: I35.0). Exclusion criteria included a history of endocarditis (ICD-10-CM: I38), aortocoronary bypass graft surgery (ICD-10-CM: Z:95.1), and the presence of prosthetic or xenogeneic heart valves (ICD-10-CM: Z95.2 and ICD-10-CM: Z95.3). Medications were assessed using ATC codes. SGLT2 inhibitor exposure was analyzed as a class effect as the current dataset does not provide sufficient detail. In the SGLT2 inhibitor cohort, the index date was the date of first SGLT2 inhibitor prescription in a patient with a documented diagnosis of non-rheumatic aortic stenosis (I35.0) recorded on or before that prescription. In the non-SGLT2 inhibitor cohort, the index date was the date of the first VA-class medication prescription (any medication class) in a patient with I35.0 and no history of SGLT2i exposure. Balanced cohorts were created using 1:1 propensity score matching (PSM) through logistic regression, employing greedy nearest neighbor matching with a caliper of 0.1 pooled standard deviations, without replacement with a caliper width of 0.1 pooled SD of the logit of the propensity score. The 0.1 caliper was chosen as a standard approach to reduce residual bias while retaining an adequate sample size. Absolute standardized mean differences (SD) were used to show the distribution of demographic and clinical data among the groups and was calculated as the difference in the means or proportions of a particular variable divided by the pooled estimate of SD for that variable. Any baseline characteristic with an SD < 10% was considered well matched.

Propensity scores were estimated using a comprehensive set of 82 baseline variables reflecting demographic characteristics, clinical comorbidities, medication use, and objective clinical measurements (Supplementary Table S1). Demographic factors included age, sex, and detailed ethnicity categories. Comorbidity variables comprised a broad range of cardiovascular, metabolic, renal, pulmonary, neurologic, hematologic, and oncologic diagnoses, as well as prior device implantation and lifestyle-related conditions. Medication variables captured exposure to major therapeutic classes relevant to cardiovascular and metabolic disease, including beta blockers, lipid-lowering agents, diuretics, calcium channel blockers, renin–angiotensin system inhibitors, antiplatelet agents, antiarrhythmic drugs, and multiple classes of glucose-lowering therapies. Laboratory and echocardiographic measurements included lipid parameters, hemoglobin A1c, body mass index, blood pressure, kidney function (eGFR), natriuretic peptides, heart rate, left ventricular ejection fraction, and aortic valve hemodynamic indices. These variables were selected a priori to ensure robust control of confounding and to optimize balance between the matched cohorts.

SMD < 10% was considered adequate balance. Outcomes were assessed in a predefined window from day 1 to day 1095 (3 years) after the index date for both cohorts. After PSM, Cox proportional hazard models were used to calculate hazard ratios (HRs) and 95% confidence intervals (95% CI) for the risk of adverse events in patients treated with SGLT2 inhibitors. AVR analyses use standard Cox proportional hazards with censoring at death. The TriNetX platform does not allow calculation or display of the number of patients at risk over time, which limits the ability to provide traditional “numbers at risk” tables. This research network has been previously used to study patients with diabetes and cardiovascular conditions [8,9] and valvular disease [10].

## 3. Results

Among 228,913 patients with AS, 28,961 (12.6%) received SGLT2 inhibitor therapy. Prior to propensity score matching, patients treated with SGLT2 inhibitors were older, more frequently male and African American, and had a higher prevalence of cardiovascular risk factors—including diabetes—as well as cardiovascular comorbidities such as heart failure, coronary artery disease, peripheral artery disease, and chronic kidney disease. Baseline characteristics after PSM are summarized in Table 1.

After matching, the median follow-up was 1.22 years (IQR 2.32). Median follow-up was 0.92 years (IQR 1.84) among SGLT2i users and 1.53 years (IQR 2.80) among non-users. The annual mortality rate was 6.15% in the SGLT2 inhibitor-treated group compared to 9.34% in the control group, consistent with rates reported in a large meta-analysis of patients with moderate AS (9%, n = 12,143) [11].

**Figure 1:**
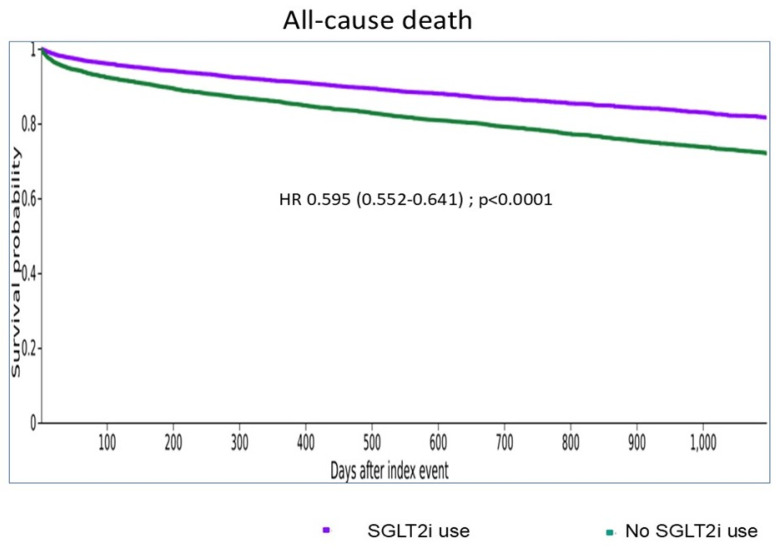
Cumulative incidence of all-cause death in degenerative aortic stenosis patients treated or not by SGLT2 inhibitors.

SGLT2 inhibitor use was associated with a significantly lower risk of all-cause mortality (HR: 0.595; 95% CI: 0.552–0.641; *p* < 0.001), cardiac arrest (HR: 0.71; 95% CI: 0.582–0.867; *p* < 0.001), TAVR (HR: 0.835; 95% CI: 0.746–0.934; *p* = 0.002), SAVR (HR: 0.514; 95% CI: 0.442–0.599; *p* < 0.001), and end-stage kidney disease (HR: 0.292; 95% CI: 0.222–0.384; *p* < 0.001) (Table 2, Figure 1 and Graphical Abstract). In contrast, no significant differences were observed between groups in the incidence of heart failure and AF.

## 4. Discussion

In this large, real-world cohort of patients with degenerative AS, treatment with SGLT2 inhibitors was independently associated with lower all-cause mortality and reduced rates of aortic valve replacement and end-stage kidney disease (ESKD), even after comprehensive adjustment for baseline comorbidities. These findings suggest that SGLT2 inhibitors may confer cardioprotective, renoprotective, and valve-modifying benefits in this high-risk population. However, the observational nature of this study precludes any inference of causal or disease-modifying activity.

While the cardiovascular benefits of SGLT2 inhibitors in heart failure are well established, emerging evidence suggests that these agents may exert therapeutic effects not only on the ventricle but also on the aortic valve—or potentially both [4,12,13,14]. SGLT2 expression has recently been identified in stenotic aortic valves, where it colocalizes with areas of calcification and monocytic infiltration and appears to contribute to pro-oxidative signaling pathways involved in valvular endothelial dysfunction, inflammation, and thrombogenicity [4]. These detrimental processes have been attenuated in vitro by SGLT2 inhibition [4].

Recent findings suggest that the ex vivo effects of SGLT2 on valvular remodeling observed in experimental tissue models may hold clinical relevance, potentially translating into measurable benefits through the deceleration of degenerative valve disease progression [4]. These insights extend the therapeutic potential of SGLT2 inhibitors beyond glycemic control and heart failure management, highlighting a possible role in the modulation of valvular pathobiology.

In addition to their established cardioprotective actions, emerging clinical evidence indicates that SGLT2 inhibitors may attenuate the progression of aortic stenosis (AS), particularly in its moderate stages. A post hoc analysis involving 458 patients receiving SGLT2 inhibitors and 11,240 untreated controls demonstrated a significantly lower risk of progression from non-severe to severe AS among treated patients, after adjustment for time-varying exposure, covariates, and competing risks (HR 0.61; 95% CI, 0.39–0.94; *p* = 0.03) over a median follow-up of 3.4 years. Notably, the protective effect appeared to intensify with longer treatment duration, with hazard ratios of 0.54, 0.48, and 0.27 for 3-, 6-, and 12-month therapy durations, respectively—suggesting a possible cumulative benefit [15]. In the present analysis, based on a large, real-world cohort of patients with degenerative AS, the use of SGLT2 inhibitors was associated not only with reduced overall mortality but also with a significant decrease in the need for transcatheter aortic valve replacement (TAVR; HR 0.835, 95% CI 0.746–0.934; *p* = 0.002) and surgical aortic valve replacement (SAVR; HR 0.514, 95% CI 0.442–0.599; *p* < 0.001), serving as surrogate indicators of slower valvular disease progression.

Supporting these observations, a retrospective analysis of 1838 adults who underwent aortic valve replacement—either transcatheter (TAVR) or surgical (SAVR)—showed a significantly lower incidence of bioprosthetic valve dysfunction among SGLT2 inhibitor users compared with non-users (HR 0.37; 95% CI, 0.18–0.78; *p* = 0.008) over a median follow-up of 4.85 years [16]. Similarly, recent findings from our large real-world cohort of TAVR recipients demonstrated that SGLT2 inhibitor therapy was independently associated with reduced all-cause mortality and lower rates of bioprosthetic valve failure, even after extensive adjustment for baseline comorbidities [10].

Apart from their crucial impact on valvular disease progression, SGLT2 inhibitors are also expected to mitigate heart failure progression, as demonstrated in the DapaTAVR trial [13] or in large multicenter cohort [17]. In contrast, no significant reduction in heart failure incidence was observed among patients treated with SGLT2 inhibitors in the present analysis, and higher rates of hospitalization for heart failure were even noted among SGLT2i users. This unexpected finding may be partly explained by residual confounding factors, including a higher prevalence of heart failure with preserved ejection fraction (HFpEF) within the SGLT2 inhibitor-treated cohort—a condition for which these agents are most commonly prescribed. Additionally, patients receiving SGLT2 inhibitors may benefit from more intensive medical follow-up, leading to earlier recognition of decompensating symptoms and consequently a lower threshold for hospital admission. This combination of baseline risk differences and patterns of healthcare utilization may therefore contribute to the observed increase in heart failure hospitalization in the SGLT2i group, rather than indicating a lack of therapeutic benefit.

Taken together, these data collectively support the concept that SGLT2 inhibitors may exert both cardioprotective [18] and valvuloprotective effects in degenerative AS. Mechanistically, these benefits may stem from improved endothelial function, modulation of oxidative stress and extracellular matrix remodeling, and suppression of inflammatory, profibrotic, and procalcifying signaling pathways [3,4,19]. However, the associations observed in this study cannot establish any causal effect on AS progression, and the absence of systematic imaging further limits mechanistic interpretation. While further prospective studies are required to establish causality, these observations open a promising therapeutic perspective for the medical management of degenerative aortic stenosis—a disease for which no pharmacologic treatment currently exists.

### Study Limitations

This study has several limitations that should be acknowledged. First, the retrospective and observational design precludes definitive causal inference, and residual confounding cannot be entirely excluded despite extensive propensity score matching across 83 clinical and demographic variables. Second, the use of electronic medical record data from the TriNetX platform introduces potential biases related to diagnostic coding accuracy, incomplete data capture, especially echocardiography data, and heterogeneity among contributing healthcare organizations. Third, the follow-up period was relatively limited (median 1.22 (IQR 2.32)), which may not fully capture the long-term effects of SGLT2 inhibitor therapy on the progression of aortic stenosis or on valvular calcification. Fourth, because echocardiographic data were only available at baseline and at follow-up for a very limited proportion of the cohort, AS severity, valve morphology, and etiology cannot be reliably captured using ICD-10 code I35.0. This code encompasses mild, moderate, and severe AS, and although baseline echocardiographic parameters were included in the matching process, residual heterogeneity in disease severity cannot be excluded. Moreover, the absence of imaging-based endpoints limits the ability to directly assess structural or functional changes in the aortic valve.

Additionally, medication exposure was determined from prescription records rather than verified patient adherence, and differences in drug type, dose, or treatment duration were not analyzed. SGLT2 inhibitor exposure was analyzed as a class effect, as the current dataset does not allow robust assessment of agent-specific or dose response relationships. Limitations in prescription granularity, dosing information, and duration of therapy preclude robust evaluation of individual agents or their specific pharmacological differences.

Dedicated competing-risk analyses (e.g., Fine Grays models) would be needed on individual-level data to precisely disentangle effects on AVR or versus death but were not feasible with the current TriNetX platform. This approach does not formally account death as a competing event; therefore, the observed reduction in AVR may partially reflect the interplay between mortality differences and treatment selection. Prevalent-user bias and residual immortal time bias cannot be completely ruled out, especially as some patients may have had AS diagnosed long before SGLT2 initiation. Finally, as most patients receiving SGLT2 inhibitors likely had concomitant diabetes mellitus or heart failure, the generalizability of these findings to non-diabetic or asymptomatic aortic stenosis populations remains uncertain. Future prospective, randomized controlled trials with longer follow-up and detailed phenotypic characterization are warranted to confirm these findings and clarify the underlying mechanisms.

## 5. Conclusions

In this large, real-world cohort of patients with aortic stenosis, treatment with sodium–glucose cotransporter 2 inhibitors was independently associated with lower all-cause mortality and reduced rates of aortic valve replacement and end-stage kidney disease, even after comprehensive adjustment for comorbidities and clinical confounders. These associations raise the possibility that SGLT2 inhibitors may have favorable effects on valve-related outcomes, although the design of this study does not allow conclusions regarding causality or direct effects on aortic stenosis progression. The underlying mechanisms may involve attenuation of oxidative stress, inflammation, and fibrosis within valvular and myocardial tissues, consistent with emerging experimental data. While these observations provide compelling evidence supporting the therapeutic potential of SGLT2 inhibition in valvular heart disease, prospective randomized studies with detailed echocardiographic and biomarker assessments are required to confirm causality and further elucidate the biological pathways involved. Collectively, our findings highlight a promising new avenue for the medical management of degenerative aortic stenosis and warrant further clinical investigation.

## Figures and Tables

**Table 1:** Baseline characteristics of patients with degenerative AS after propensity score matching.

	AS, SGLT2 Inhibitors	AS, No SGLT2 Inhibitors	Std Diff. (%)
	(n = 10,912)	(n = 10,912)	
**Demographics**			
Age (years), mean ± SD	73.4 +/− 11.6	73.6 +/− 11.5	1.8
Men, n (%)	5933 (54.4%)	5947 (54.5%)	0.3
White, n (%)	7069 (64.8%)	7031 (64.4%)	0.7
Black or African American, n (%)	941 (8.6%)	938 (8.6%)	0.1
Socioeconomic and psychosocial circumstances, n (%)	224 (2.1%)	210 (1.9%)	0.9
**Risk factors**			
Hypertension, n (%)	7116 (65.2%)	7029 (64.4%)	1.7
Diabetes mellitus, n (%)	5631 (51.6%)	5786 (53%)	2.8
Smoker, n (%)	1688 (15.5%)	1685 (15.4%)	0.1
Overweight or obesity, n (%)	2378 (21.8%)	2405 (22%)	0.6
Dyslipidemia, n (%)	6621 (60.7%)	6598 (60.5%)	0.4
**Cardiovascular comorbidities**			
Heart failure, n (%)	4731 (43.4%)	4724 (43.3%)	0.1
Coronary artery disease, n (%)	4759 (43.6%)	4775 (43.8%)	0.3
Myocardial infarction, n (%)	1127 (10.3%)	1116 (10.2%)	0.3
Ischemic stroke, n (%)	548 (5%)	558 (5.1%)	0.4
Atrial fibrillation or flutter, n (%)	3182 (29.2%)	3260 (29.9%)	1.6
Peripheral vascular disease, n (%)	816 (7.5%)	820 (7.5%)	0.1
**Non-cardiovascular comorbidities**			
Kidney disease, n (%)	3422 (31.4%)	3406 (31.2%)	0.3
COPD, n (%)	1271 (11.6%)	1293 (11.8%)	0.6
Sleep apnea syndrome, n (%)	1579 (14.5%)	1561 (14.3%)	0.5
Previous cancer, n (%)	2192 (20.1%)	2157 (19.8%)	0.8
Cognitive impairment, n (%)	93 (0.9%)	81 (0.7%)	1.2
**Laboratory tests and examinations**			
Body mass index (kg/m^2^), mean ± SD	30.6 +/− 7.6	30.5 +/− 7.5	0.2
Total cholesterol (mg/dL), mean ± SD	155.5 +/− 47.0	157.8 +/− 45.5	5
LDL cholesterol (mg/dL), mean ± SD	83.2 +/− 36.5	86.4 +/− 34.8	9
HDL cholesterol (mg/dL), mean ± SD	46.1 +/− 17.8	46.6 +/− 17.4	3.1
Triglyceride (mg/dL), mean ± SD	138.7 +/− 98.2	132.4 +/− 91.1	6.7
HbA1c ≥ 6%, n (%)	3058 (28%)	2961 (27.1%)	2
Estimated GFR (MDRD, ml/min), mean ± SD	62.3 +/− 25.7	60.3 +/− 27.2	7.6
BNP, ng/L, mean ± SD	1259.4 +/− 3194.1	1056.7 +/− 2706.7	6.8
NT-proBNP, ng/L, mean ± SD	4869.7 +/− 8035.9	5223.1 +/− 8777.1	4.2
LVEF, mean ± SD	54.9 +/− 14.5	54.1 +/− 16.0	5.3
**Medications**			
Beta blockers, n (%)	6341 (58.1%)	6386 (58.5%)	0.8
Calcium channel blockers, n (%)	3845 (35.2%)	3807 (34.9%)	0.7
ACE Inhibitors, n (%)	3038 (27.8%)	3027 (27.7%)	0.2
Angiotensin II Inhibitors, n (%)	3264 (29.9%)	3246 (29.7%)	0.4
ARNI, n (%)	327 (3%)	315 (2.9%)	0.7
MRA, n (%)	1614 (14.8%)	1639 (15%)	0.6
Digitalis glycosides, n (%)	402 (3.7%)	404 (3.7%)	0.1
Diuretics, n (%)	6306 (57.8%)	6367 (58.3%)	1.1
Lipid lowering drugs, n (%)	7200 (66%)	7228 (66.2%)	0.5
Insulin, n (%)	2893 (26.5%)	2889 (26.5%)	0.1
Metformin, n (%)	2711 (24.8%)	2794 (25.6%)	1.8
Sulfonylureas, n (%)	1323 (12.1%)	1344 (12.3%)	0.6
GLP-1 receptor agonists, n (%)	402 (3.7%)	385 (3.5%)	0.8
DPP4 inhibitors, n (%)	882 (8.1%)	889 (8.1%)	0.2
SGLT2 inhibitors, n (%)	10,912 (100%)	0 (0%)	0
Thiazolidinediones, n (%)	230 (2.1%)	261 (2.4%)	1.9
Antiplatelet therapy, n (%)	5180 (47.5%)	5238 (48%)	1.1
Anticoagulant, n (%)	1376 (12.6%)	1419 (13%)	1.2

**Table 2:** Clinical outcomes.

Clinical Outcomes (Median FU 1.22, IQR 2.32)				
	Number of Events (Yearly Rate, %)		Hazard Ratio (95% CI)	*p* Value
Death	1045 (6.15)	2084 (9.34)	0.595 (0.552–0.641)	<0.0001
SAVR	251 (1.28)	499 (1.90)	0.514 (0.442–0.599)	<0.0001
TAVR	554 (2.81)	698 (2.89)	0.835 (0.746–0.934)	0.002
Ischemic stroke or thromboembolism	328 (2.25)	430 (2.29)	0.914 (0.791–1.056)	0.22
Acute MI	152 (0.84)	205 (0.87)	0.822 (0.666–1.015)	0.07
Incident HF	1138 (10.38)	1256 (9.64)	1.023 (0.943–1.108)	0.59
Hospitalization for HF	970 (4.01)	706 (2.76)	1.436 (1.303–1.582)	<0.0001
Incident AF	896 (6.97)	1087 (7.03)	0.918 (0.840–1.003)	0.06
Cardiac arrest	159 (0.82)	255 (1.21)	0.71 (0.582–0.867)	0.001
VT/VF	485 (2.68)	529 (2.37)	1.02 (0.901–1.155)	0.75
ESKD	66 (0.40)	243 (1.00)	0.292 (0.222–0.384)	<0.0001

## Data Availability

The data presented in this study are available on request from the corresponding author.

## Supplementary Materials

The following supporting information can be downloaded at: https://www.mdpi.com/article/10.3390/jcm15020714/s1.

## Abbreviations

The following abbreviations are used in this manuscript:

ACE	Angiotensin-Converting Enzyme
AF	Atrial fibrillation
AKI	Acute Kidney Injury (if needed; appears indirectly via kidney outcomes)
ARNI	Angiotensin Receptor–Neprilysin Inhibitor
AS	Aortic stenosis
AT1R	Angiotensin II Type-1 Receptor
ATC	Anatomical Therapeutic Chemical Classification
AVA	Aortic valve area
AVR	Aortic valve replacement
BNP	B-type natriuretic peptide
CI	Confidence interval
CKD	Chronic kidney disease
CM	Clinical Modification (as in ICD-10-CM)
COPD	Chronic Obstructive Pulmonary Disease
DPP-4	Dipeptidyl Peptidase-4
EF	Ejection fraction (used as LVEF)
EMR	Electronic medical record
ESKD	End-stage kidney disease
FXa	Activated factor X
GFR	Glomerular Filtration Rate
GLP-1	Glucagon-Like Peptide-1
HbA1c	Hemoglobin A1c
HF	Heart failure
HFpEF	Heart failure with preserved ejection fraction
HR	Hazard ratio
ICD-9/ICD-10-CM	International Classification of Diseases, 9th/10th Revision, Clinical Modification
IQR	Interquartile Range
IV	Interventricular
LF/LG AS	Low-flow, low-gradient aortic stenosis
LVEF	Left ventricular ejection fraction
MI	Myocardial infarction
MRA	Mineralocorticoid Receptor Antagonist
MDRD	Modification of Diet in Renal Disease (formula for GFR)
NT-proBNP	N-Terminal pro-B-type natriuretic peptide
PSM	Propensity score matching
SAVR	Surgical aortic valve replacement
SD	Standard deviation
SGLT2	Sodium–glucose cotransporter 2
SGLT2i	SGLT2 inhibitor
SMD	Standardized mean difference
T2DM	Type 2 diabetes mellitus
TAVR	Transcatheter aortic valve replacement
VEC	Valvular endothelial cell
Vmax	Peak Aortic Jet Velocity
VT/VF	Ventricular Tachycardia/Ventricular Fibrillation

## References

[1] Usman M.S., Siddiqi T.J., Anker S.D., Bakris G.L., Bhatt D.L., Filippatos G., Fonarow G.C., Greene S.J., Januzzi J.L., Khan M.S. (2023). Effect of SGLT2 Inhibitors on Cardiovascular Outcomes Across Various Patient Populations. J. Am. Coll. Cardiol..

[2] Goldberg L.R. (2021). The Pleiotropic Effects of SGLT2 Inhibitors: Remodeling the Treatment of Heart Failure. J. Am. Coll. Cardiol..

[3] Mroueh A., Algara-Suarez P., Fakih W., Gong D.S., Matsushita K., Park S.H., Amissi S., Auger C., Kauffenstein G., Meyer N. (2024). SGLT2 expression in human vasculature and heart correlates with low-grade inflammation and causes eNOS-NO/ROS imbalance. Cardiovasc. Res..

[4] Hmadeh S., Trimaille A., Matsushita K., Marchandot B., Carmona A., Zobairi F., Sato C., Kindo M., Hoang T.M., Toti F. (2024). Human Aortic Stenotic Valve-Derived Extracellular Vesicles Induce Endothelial Dysfunction and Thrombogenicity Through AT1R/NADPH Oxidases/SGLT2 Pro-Oxidant Pathway. JACC Basic. Transl. Sci..

[5] Trimaille A., Hmadeh S., Matsushita K., Marchandot B., Kauffenstein G., Morel O. (2023). Aortic stenosis and the haemostatic system. Cardiovasc. Res..

[6] Scisciola L., Paolisso P., Belmonte M., Gallinoro E., Delrue L., Taktaz F., Fontanella R.A., Degrieck I., Pesapane A., Casselman F. (2024). Myocardial sodium-glucose cotransporter 2 expression and cardiac remodelling in patients with severe aortic stenosis: The BIO-AS study. Eur. J. Heart Fail..

[7] Trimaille A., Hmadeh S., Kikuchi S., Mroueh A., Carmona A., Marchandot B., Beras F., Truong D.P., Vu M.C., Granier A. (2025). Detrimental effects of plasma from patients with severe aortic stenosis on valvular endothelial cells: Role of proinflammatory cytokines and factor Xa. J. Am. Heart Assoc..

[8] Bucci T., Alam U., Fauchier G., Lochon L., Bisson A., Ducluzeau P.H., Lip G.Y.H., Fauchier L. (2025). GLP-1 receptor agonists and cardiovascular events in metabolically healthy or unhealthy obesity. Diabetes Obes. Metab..

[9] Bucci T., Gerra L., Lam S.H.M., Argyris A.A., Boriani G., Proietti R., Bisson A., Fauchier L., Lip G.Y.H. (2024). Risk of death and thrombosis in patients admitted to the emergency department with supraventricular tachycardias. Heart Rhythm. Off. J. Heart Rhythm. Soc..

[10] Morel O., Granier A., Lochon L., Trimaille A., Bisson A., Marchandot B., Bernard A., Fauchier L. (2025). Association of SGLT2 Inhibitors with Mortality and Bioprosthesis Valve Failure After TAVR: A Propensity-Matched Cohort Study. J. Clin. Med..

[11] Coisne A., Scotti A., Latib A., Montaigne D., Ho E.C., Ludwig S., Modine T., Genereux P., Bax J.J., Leon M.B. (2022). Impact of Moderate Aortic Stenosis on Long-Term Clinical Outcomes: A Systematic Review and Meta-Analysis. JACC Cardiovasc. Interv..

[12] Kamperidis V., Anastasiou V., Ziakas A. (2025). Could SGLT2 inhibitors improve outcomes in patients with heart failure and significant valvular heart disease? Need for action. Heart Fail. Rev..

[13] Raposeiras-Roubin S., Amat-Santos I.J., Rossello X., Gonzalez Ferreiro R., Gonzalez Bermudez I., Lopez Otero D., Nombela-Franco L., Gheorghe L., Diez J.L., Baladron Zorita C. (2025). Dapagliflozin in Patients Undergoing Transcatheter Aortic-Valve Implantation. N. Engl. J. Med..

[14] Sato R., Sakai K., Kameshima S. (2025). Dapagliflozin inhibits TGF-beta-induced transdifferentiation of valvular interstitial cells and mitral valvular degeneration. J. Mol. Med..

[15] Shah T., Zhang Z., Shah H., Fanaroff A.C., Nathan A.S., Parise H., Lutz J., Sugeng L., Bellumkonda L., Redfors B. (2025). Effect of Sodium-Glucose Cotransporter-2 Inhibitors on the Progression of Aortic Stenosis. JACC Cardiovasc. Interv..

[16] Abbas M.T., Awad K., Farina J.M., Tamarappoo B.K., Lee K.S., Lester S.J., Alsidawi S., Sell-Dottin K.A., Ayoub C., Arsanjani R. (2025). The Association Between Sodium-Glucose Cotransporter 2 Inhibitors and Bioprosthetic Aortic Valve Degeneration. JACC Adv..

[17] Obeidat O., Alayyat A., Naser A., Ghanem F., Jabri A., Brankovic M., Jiao T., Ruzieh M., Haddad A., Alexy T. (2025). Impact of SGLT2 inhibitors on long-term outcomes in TAVI patients with heart failure: A propensity-matched analysis. Rev. Esp. De Cardiol..

[18] Castro Conde A., Marzal Martin D., Campuzano Ruiz R., Fernandez Olmo M.R., Morillas Arino C., Gomez Doblas J.J., Gorriz Teruel J.L., Mazon Ramos P., Garcia-Moll Marimon X., Soler Romeo M.J. (2023). Comprehensive Cardiovascular and Renal Protection in Patients with Type 2 Diabetes. J. Clin. Med..

[19] Goes-Santos B.R., Castro P.C., Girardi A.C.C., Antunes-Correa L.M., Davel A.P. (2025). Vascular effects of SGLT2 inhibitors: Evidence and mechanisms. Am. J. Physiol. Cell Physiol..

