# Supplementary material for: Assessing Potential Valve-Preserving Effects of SGLT2 Inhibitors in Degenerative Aortic Stenosis: A Propensity-Matched Study
Source: J Clin Med. 2026 Jan 15;15(2):714. doi: 10.3390/jcm15020714 (PMC12842084; doi:10.3390/jcm15020714)
Supplement: Supplementary file 1 [file jcm-15-00714-s001.zip › jcm-4005166-supplementary.pdf]

Table S1.

Cohort 1 (N = 10,912) and cohort 2 (N = 10,912) characteristics after propensity score matching

Demographics

	Cohort			Mean ± SD	Patients	% of Cohort	P-Value	Std diff.
1	1<br>2	AI	Age at Index	73.4 +/- 11.6<br>73.6 +/- 11.5	10,912<br>10,912	100%<br>100%	0.195	0.018
2	1<br>2	2106-3	White		7,069<br>7,031	64.8%<br>64.4%	0.591	0.007
3	1<br>2	UNK	Unknown Race		2,093<br>2,144	19.2%<br>19.6%	0.383	0.012
4	1<br>2	2135-2	Hispanic or Latino		491<br>487	4.5%<br>4.5%	0.896	0.002
5	1<br>2	2054-5	Black or African American		941<br>938	8.6%<br>8.6%	0.942	0.001
6	1<br>2	M	Male		5,933<br>5,947	54.4%<br>54.5%	0.849	0.003
7	1<br>2	2028-9	Asian		441<br>409	4.0%<br>3.7%	0.263	0.015

Diagnosis

	Cohort			Mean ± SD	Patients	% of Cohort	P-Value	Std diff.
8	1<br>2	I10	Essential (primary) hypertension		7,116<br>7,029	65.2%<br>64.4%	0.218	0.017
9	1<br>2	I25	Chronic ischemic heart disease		4,759<br>4,775	43.6%<br>43.8%	0.827	0.003
10	1<br>2	I21	Acute myocardial infarction		1,127<br>1,116	10.3%<br>10.2%	0.806	0.003
11	1<br>2	I63	Cerebral infarction		548<br>558	5.0%<br>5.1%	0.758	0.004
12	1<br>2	I61	Nontraumatic intracerebral hemorrhage		68<br>72	0.6%<br>0.7%	0.734	0.005
13	1<br>2	E08-E13	Diabetes mellitus		5,631<br>5,786	51.6%<br>53.0%	0.036	0.028
14	1<br>2	E65-E68	Overweight, obesity and other hyperalimentation		2,378<br>2,405	21.8%<br>22.0%	0.659	0.006
15	1<br>2	E00-E07	Disorders of thyroid gland		1,825<br>1,755	16.7%<br>16.1%	0.201	0.017
16	1<br>2	E40-E46	Malnutrition		319<br>329	2.9%<br>3.0%	0.690	0.005
17	1<br>2	E78	Disorders of lipoprotein metabolism and other lipidemias		6,621<br>6,598	60.7%<br>60.5%	0.750	0.004
18	1<br>2	E85	Amyloidosis		64<br>69	0.6%<br>0.6%	0.664	0.006
19	1<br>2	I50	Heart failure		4,731<br>4,724	43.4%<br>43.3%	0.924	0.001
20	1<br>2	I34	Nonrheumatic mitral valve disorders		2,003<br>1,931	18.4%<br>17.7%	0.205	0.017
21	1<br>2	I44	Atrioventricular and left bundle-branch block		1,275<br>1,285	11.7%<br>11.8%	0.833	0.003
22	1<br>2	I44.7	Left bundle-branch block, unspecified		464<br>486	4.3%<br>4.5%	0.465	0.010
23	1<br>2	N17-N19	Acute kidney failure and chronic kidney disease		3,422<br>3,406	31.4%<br>31.2%	0.815	0.003

24	1<br>2	J44.9	Chronic obstructive pulmonary disease, unspecified	1,271<br>1,293	11.6%<br>11.8%	0.644	0.006
25	1<br>2	J00-J99	Diseases of the respiratory system	4,652<br>4,650	42.6%<br>42.6%	0.978	<0.001
26	1<br>2	G47.3	Sleep apnea	1,579<br>1,561	14.5%<br>14.3%	0.728	0.005
27	1<br>2	I73.9	Peripheral vascular disease, unspecified	816<br>820	7.5%<br>7.5%	0.918	0.001
28	1<br>2	Z87.891	Personal history of nicotine dependence	1,688<br>1,685	15.5%<br>15.4%	0.955	0.001
29	1<br>2	I49.5	Sick sinus syndrome	339<br>328	3.1%<br>3.0%	0.665	0.006
30	1<br>2	I34.0	Nonrheumatic mitral (valve) insufficiency	1,794<br>1,724	16.4%<br>15.8%	0.198	0.017
31	1<br>2	I35.1	Nonrheumatic aortic (valve) insufficiency	2,980<br>2,803	27.3%<br>25.7%	0.007	0.037
32	1<br>2	I05	Rheumatic mitral valve diseases	272<br>243	2.5%<br>2.2%	0.196	0.018
33	1<br>2	I42.0	Dilated cardiomyopathy	243<br>231	2.2%<br>2.1%	0.577	0.008
34	1<br>2	I48	Atrial fibrillation and flutter	3,182<br>3,260	29.2%<br>29.9%	0.247	0.016
35	1<br>2	Z95.0	Presence of cardiac pacemaker	590<br>593	5.4%<br>5.4%	0.929	0.001
36	1<br>2	Z95.810	Presence of automatic (implantable) cardiac defibrillator	260<br>251	2.4%<br>2.3%	0.687	0.005
37	1<br>2	F10.1	Alcohol abuse	150<br>157	1.4%<br>1.4%	0.687	0.005
38	1<br>2	C00-D49	Neoplasms	2,192<br>2,157	20.1%<br>19.8%	0.553	0.008
39	1<br>2	G30	Alzheimer's disease	93<br>81	0.9%<br>0.7%	0.361	0.012
40	1<br>2	E10	Type 1 diabetes mellitus	290<br>275	2.7%<br>2.5%	0.523	0.009
41	1<br>2	F40-F48	Anxiety, dissociative, stress-related, somatoform and other nonpsychotic mental disorders	1,236<br>1,197	11.3%<br>11.0%	0.402	0.011
42	1<br>2	F30-F39	Mood [affective] disorders	1,171<br>1,168	10.7%<br>10.7%	0.948	0.001
43	1<br>2	Z79.01	Long term (current) use of anticoagulants	1,376<br>1,419	12.6%<br>13.0%	0.384	0.012
44	1<br>2	I44.2	Atrioventricular block, complete	291<br>292	2.7%<br>2.7%	0.967	0.001
45	1<br>2	I42	Cardiomyopathy	1,266<br>1,217	11.6%<br>11.2%	0.296	0.014
46	1<br>2	Z55-Z65	Persons with potential health hazards related to socioeconomic and psychosocial circumstances	224<br>210	2.1%<br>1.9%	0.497	0.009
47	1<br>2	I38	Endocarditis, valve unspecified	0<br>0	0%<br>0%	--	--

	Cohort			Mean ± SD	Patients	% of Cohort	P-Value	Std diff.
48	1<br>2	D64.9	Anemia, unspecified		1,606<br>1,555	14.7%<br>14.3%	0.327	0.013
**Medication**								
	Cohort			Mean ± SD	Patients	% of Cohort	P-Value	Std diff.
49	1<br>2	CV100	BETA BLOCKERS/RELATED		6,341<br>6,386	58.1%<br>58.5%	0.537	0.008
50	1<br>2	CV350	ANTILIPEMIC AGENTS		7,200<br>7,228	66.0%<br>66.2%	0.689	0.005
51	1<br>2	CV700	DIURETICS		6,306<br>6,367	57.8%<br>58.3%	0.403	0.011
52	1<br>2	CV200	CALCIUM CHANNEL BLOCKERS		3,845<br>3,807	35.2%<br>34.9%	0.590	0.007
53	1<br>2	CV800	ACE INHIBITORS		3,038<br>3,027	27.8%<br>27.7%	0.868	0.002
54	1<br>2	CV805	ANGIOTENSIN II INHIBITOR		3,264<br>3,246	29.9%<br>29.7%	0.790	0.004
55	1<br>2	CV050	DIGITALIS GLYCOSIDES		402<br>404	3.7%<br>3.7%	0.943	0.001
56	1<br>2	A10A	INSULINS AND ANALOGUES		2,893<br>2,889	26.5%<br>26.5%	0.951	0.001
57	1<br>2	A10BA	Biguanides		2,711<br>2,794	24.8%<br>25.6%	0.196	0.018
58	1<br>2	A10BB	Sulfonylureas		1,323<br>1,344	12.1%<br>12.3%	0.664	0.006
59	1<br>2	A10BJ	Glucagon-like peptide-1 (GLP-1) analogues		402<br>385	3.7%<br>3.5%	0.537	0.008
60	1<br>2	A10BH	Dipeptidyl peptidase 4 (DPP-4) inhibitors		882<br>889	8.1%<br>8.1%	0.862	0.002
61	1<br>2	A10BG	Thiazolidinediones		230<br>261	2.1%<br>2.4%	0.157	0.019
62	1<br>2	BL117	PLATELET AGGREGATION INHIBITORS		5,180<br>5,238	47.5%<br>48.0%	0.432	0.011
63	1<br>2	703	amiodarone		608<br>616	5.6%<br>5.6%	0.814	0.003
64	1<br>2	1656328	sacubitril		327<br>315	3.0%<br>2.9%	0.631	0.007
65	1<br>2	CV704	POTASSIUM SPARING/COMBINATIONS DIURETICS		1,614<br>1,639	14.8%<br>15.0%	0.635	0.006
**Laboratory**								
	Cohort			Mean ± SD	Patients	% of Cohort	P-Value	Std diff.
66	1<br>2	9000	Cholesterol [Mass/volume] in Serum or Plasma	155.5 +/- 47.0<br>157.8 +/- 45.5	3,989<br>3,809	36.6%<br>34.9%	0.027	0.050
	1<br>2		0 - 200 mg/dL		3,548<br>3,356	32.5%<br>30.8%	0.005	0.038
	1<br>2		200 - 0 mg/dL		930<br>877	8.5%<br>8.0%	0.193	0.018
67	1<br>2	9002	Cholesterol in LDL [Mass/volume] in Serum or Plasma	83.2 +/- 36.5<br>86.4 +/- 34.8	4,032<br>3,872	37.0%<br>35.5%	<0.001	0.090
	1<br>2		0 - 100 mg/dL		3,108<br>2,953	28.5%<br>27.1%	0.019	0.032
	1<br>2		100 - 0 mg/dL		1,538<br>1,474	14.1%<br>13.5%	0.209	0.017

68	1<br>2	9001	Cholesterol in HDL [Mass/volume] in Serum or Plasma	46.1 +/- 17.8<br>46.6 +/- 17.4	4,059<br>3,799	37.2%<br>34.8%	0.173	0.031
	1<br>2		0 - 40 mg/dL		1,638<br>1,578	15.0%<br>14.5%	0.252	0.016
	1<br>2		40 - 0 mg/dL		2,972<br>2,777	27.2%<br>25.4%	0.003	0.041
69	1<br>2	9004	Triglyceride [Mass/volume] in Serum, Plasma or Blood	138.7 +/- 98.2<br>132.4 +/- 91.1	4,077<br>3,854	37.4%<br>35.3%	0.003	0.067
	1<br>2		0 - 0 mg/dL		4,077<br>3,854	37.4%<br>35.3%	0.002	0.042
70	1<br>2	9037	Hemoglobin A1c/Hemoglobin.total in Blood	7.1 +/- 1.8<br>6.8 +/- 1.7	4,238<br>3,952	38.8%<br>36.2%	<0.001	0.185
	1<br>2		0 - 6 %		1,741<br>1,710	16.0%<br>15.7%	0.565	0.008
	1<br>2		6 - 0 %		3,058<br>2,961	28.0%<br>27.1%	0.142	0.020
71	1<br>2	9083	BMI	30.6 +/- 7.6<br>30.5 +/- 7.5	6,388<br>7,251	58.5%<br>66.4%	0.925	0.002
	1<br>2		0 - 25 kg/m2		1,847<br>1,761	16.9%<br>16.1%	0.117	0.021
	1<br>2		25 - 30 kg/m2		2,627<br>2,571	24.1%<br>23.6%	0.374	0.012
	1<br>2		30 - 35 kg/m2		2,234<br>2,210	20.5%<br>20.3%	0.687	0.005
	1<br>2		35 - 0 kg/m2		1,845<br>1,880	16.9%<br>17.2%	0.529	0.009
	1<br>2	9085	Blood Pressure, Systolic	126.2 +/- 21.5<br>122.0 +/- 25.8	7,536<br>8,091	69.1%<br>74.1%	<0.001	0.180
72	1<br>2		0 - 110 mm[Hg]		3,247<br>3,157	29.8%<br>28.9%	0.181	0.018
	1<br>2		110 - 0 mm[Hg]		7,355<br>7,294	67.4%<br>66.8%	0.379	0.012
	1<br>2	9086	Blood Pressure, Diastolic	69.1 +/- 13.1<br>64.9 +/- 14.7	7,522<br>8,081	68.9%<br>74.1%	<0.001	0.305
73	1<br>2		0 - 70 mm[Hg]		5,664<br>5,562	51.9%<br>51.0%	0.167	0.019
	1<br>2		70 - 0 mm[Hg]		6,646<br>6,549	60.9%<br>60.0%	0.179	0.018
74	1<br>2	8001	Glomerular filtration rate/1.73 sq M.predicted [Volume Rate/Area] in Serum, Plasma or Blood by Creatinine-based formula (MDRD)	62.3 +/- 25.7<br>60.3 +/- 27.2	7,016<br>7,504	64.3%<br>68.8%	<0.001	0.076
	1<br>2		0 - 60 mL/min/{1.73_m2}		4,497<br>4,370	41.2%<br>40.0%	0.080	0.024
	1<br>2		60 - 90 mL/min/{1.73_m2}		4,293<br>4,205	39.3%<br>38.5%	0.222	0.017
	1<br>2		90 - 0 mL/min/{1.73_m2}		1,892<br>1,853	17.3%<br>17.0%	0.484	0.009
75	1<br>2	9003	Natriuretic peptide B [Mass/volume] in Serum, Plasma or Blood	1259.4 +/- 3194.1<br>1056.7 +/- 2706.7	1,739<br>1,800	15.9%<br>16.5%	0.041	0.068

	1<br>2		0 - 1000 pg/mL		1,364<br>1,351	12.5%<br>12.4%	0.790	0.004
	1<br>2		1000 - 1500 pg/mL		215<br>202	2.0%<br>1.9%	0.520	0.009
	1<br>2		1500 - 0 pg/mL		378<br>345	3.5%<br>3.2%	0.212	0.017
76	1<br>2	9072	Natriuretic peptide.B prohormone N-Terminal [Mass/volume] in Serum, Plasma or Blood	4869.7 +/- 8035.9<br>5223.1 +/- 8777.1	1,675<br>1,675	15.4%<br>15.4%	0.224	0.042
	1<br>2		0 - 5000 pg/mL		1,275<br>1,283	11.7%<br>11.8%	0.866	0.002
	1<br>2		5000 - 0 pg/mL		539<br>518	4.9%<br>4.7%	0.508	0.009
	1<br>2	2003	Left Ventricular Ejection Fraction (LVEF) (%)	54.9 +/- 14.5<br>54.1 +/- 16.0	797<br>792	7.3%<br>7.3%	0.294	0.053
77	1<br>2		0 - 50 %		220<br>233	2.0%<br>2.1%	0.537	0.008
	1<br>2		50 - 60 %		250<br>224	2.3%<br>2.1%	0.227	0.016
	1<br>2		60 - 0 %		441<br>427	4.0%<br>3.9%	0.628	0.007
	1<br>2	9074	Heart rate	75.6 +/- 16.5<br>72.8 +/- 15.7	5,299<br>5,974	48.6%<br>54.7%	<0.001	0.172
78	1<br>2		0 - 70 /min		3,204<br>3,199	29.4%<br>29.3%	0.941	0.001
	1<br>2		70 - 0 /min		4,512<br>4,482	41.3%<br>41.1%	0.680	0.006
	1<br>2	29430-6	Interventricular septum Thickness during diastole by US 2D	11 +/- 2.6<br>9.6 +/- 5.0	10<br>14	0.1%<br>0.1%	0.446	0.338
79	1<br>2		0 - 0 mm		10<br>14	0.1%<br>0.1%	0.414	0.011
80	1<br>2	79964-3	Aortic valve Maximum blood flow velocity during systole by US.doppler	233.3 +/- 126.5<br>268.8 +/- 105.9	128<br>127	1.2%<br>1.2%	0.016	0.304
	1<br>2		50 - 250 cm/s		58<br>62	0.5%<br>0.6%	0.714	0.005
	1<br>2		250 - 0 cm/s		55<br>67	0.5%<br>0.6%	0.276	0.015
81	1<br>2	79958-5	Aortic valve Effective orifice area during systole by US.doppler+Calculated by continuity VTI	1.5 +/- 0.8<br>1.4 +/- 0.6	27<br>38	0.2%<br>0.3%	0.584	0.135
	1<br>2		0.20 - 1.20 cm2		15<br>17	0.1%<br>0.2%	0.723	0.005
	1<br>2		1.20 - 0 cm2		14<br>22	0.1%<br>0.2%	0.182	0.018
	1<br>2	79968-4	Interventricular septum Thickness at end diastole by US.M-mode	1.2 +/- 0.2<br>1.2 +/- 0.2	65<br>85	0.6%<br>0.8%	0.947	0.011
82	1<br>2		0.50 - 1.20 cm		46<br>54	0.4%<br>0.5%	0.423	0.011
	1<br>2		1.20 - 0 cm		33<br>33	0.3%<br>0.3%	1	<0.001
